# Targeted Nrf2 activation improves vascular endothelial function with aging and prevents vascular dysfunction following disuse in younger and older adults

**DOI:** 10.1016/j.freeradbiomed.2026.05.282

**Published:** 2026-05-07

**Authors:** Nicholas A. Carlini, Brady E. Hanson, Bradley A. Ruple, Jesse C. Craig, Caitlin C. Fermoyle, Alec I. McKenzie, Angela V. Bisconti, Edward Z. Pelka, Mark A. Supiano, Eric Tuday, Russell S. Richardson, Micah J. Drummond, Namakkal S. Rajasekaran, Ryan M. Broxterman, Joel D. Trinity

**Affiliations:** a Department of Internal Medicine, Division of Geriatrics, University of Utah, Salt Lake City, UT, United States; b Geriatric Research, Education, and Clinical Center, George E. Wahlen Department of Veterans Affairs Medical Center, Salt Lake City, UT, United States; c Faculty of Medicine and Health, University of Sydney, Australia; d Department of Nutrition and Integrative Physiology, University of Utah, Salt Lake City, UT, United States; e Division of Cardiology, Department of Internal Medicine, The University of Utah School of Medicine, Salt Lake City, UT, United States; f Department of Physical Therapy and Athletic Training, University of Utah, Salt Lake City, UT, United States; g Molecular Medicine Program, University of Utah, Salt Lake City, UT, United States; h Cardiac Aging and Redox Signaling Laboratory, Molecular and Cellular Pathology, Department of Pathology/Center for Free Radical Biology, University of Alabama at Birmingham, Birmingham, AL, United States

**Keywords:** Physical inactivity, Vascular function, Redox balance

## Abstract

Aging and physical inactivity/disuse contribute to cardiovascular disease, which is attributable, in part, to vascular endothelial dysfunction associated with impaired nuclear-factor erythroid 2-related factor 2 (Nrf2) signaling. However, the ability to augment Nrf2 activation and its effects on age- and disuse-related vascular dysfunction in humans remains limited. In a double-blind, randomized, placebo-controlled study design 20 younger adults (8M/12F, age 27 ± 7 y) and 24 older adults (12M/12F, age 67 ± 8 y) were randomized to receive either placebo or PB125 (200 mg/day, Nrf2 activator) across three visits: baseline, 2 weeks of supplementation, and following either 5 days of bed rest (old) or 2 weeks of limb immobilization (young). Brachial and popliteal artery flow-mediated dilation (FMD), passive leg movement-induced (PLM) leg blood flow (LBF) and vascular conductance (LVC) and serum antioxidant status (superoxide dismutase, SOD) were assessed at each timepoint. Compared to placebo, 2 weeks of PB125 increased brachial (PB125: 2.8 ± 1.5 to 3.8 ± 1.3%, p < 0.0001) and popliteal FMD (PB125: 1.5 ± 1.5 to 2.3 ± 1.4%, p = 0.033) in older, but not younger adults (both, p > 0.27). During bed rest, PB125 preserved brachial (3.8 ± 1.3 to 3.6 ± 1.4%, p = 0.543) and popliteal FMD (2.3 ± 1.4 to 2.7 ± 1.4%, p = 0.269), LVC (1.1 ± 0.8 to 1.1 ± 1.5 AUC, p = 0.530) and circulating SOD concentrations (28.3 ± 7.2 to 28.6 ± 8.6 U/mL, p = 0.888) in older adults compared to placebo (all, p < 0.05). Following limb immobilization, PB125 preserved popliteal FMD (4.4 ± 2.8 to 3.4 ± 1.8%, p = 0.302) in younger adults compared to placebo (p < 0.05). These findings demonstrate targeted Nrf2 activation with PB125 improves age-related vascular endothelial dysfunction while preserving vascular function and antioxidant capacity following periods of disuse.

## 1. Introduction

Vascular endothelial dysfunction, a key antecedent of increased cardiovascular disease (CVD) risk, progressively worsens with advancing age [1] and is exacerbated by physical inactivity and disuse [1-4]. Disuse in older adults, commonly experienced during hospitalization following injury, illness, or surgery poses a substantial health risk [5,6] as rapid losses in vascular function are compounded by age-related vascular dysfunction [7]. In younger adults, disuse in the form of limb immobilization results in a rapid decrease in vascular function that mimics an accelerated vascular aging response. Identifying mechanisms driving vascular endothelial dysfunction and strategies to improve vascular function during aging and prevent vascular dysfunction during disuse is critical for lowering future CVD risk and minimizing the impact of disuse.

One such mechanism contributing to age- and disuse-related vascular endothelial dysfunction is excess production of reactive oxygen species (ROS) leading to oxidative stress. Multiple sources, including augmented mitochondrial superoxide ($O_{2}^{-}$) production [8,9] and increased nicotinamide adenine dinucleotide phosphate (NADPH) oxidase (NOX) activity, contribute to $O_{2}^{-}$ production that decreases nitric oxide (NO) synthase bioactivity and NO bioavailability [10,11]. Ultimately, these processes are fundamental in the regulation of vascular function. Therefore, given the redundancy in potential sources of oxidative stress, targeting cellular and molecular pathways that control redox homeostasis is paramount for the identification of novel therapeutic strategies to mitigate age- and disuse-related vascular dysfunction.

Nuclear factor erythroid 2-related factor (Nrf2) is a central regulator of redox homeostasis that responds to oxidative stress and governs the induction of endogenous antioxidants through the antioxidant response element [12-14]. Aging is associated with impaired Nrf2 signaling that impairs the ability to respond to acute increases or chronic elevations in oxidative stress thus diminishing the induction of Nrf2-related antioxidant genes and downstream endogenous antioxidants [15-18]. Impaired Nrf2 signaling has also been linked to impaired endothelial function [17,19,20]. Current *in vitro* and *ex vivo* evidence demonstrates activation of Nrf2 with the botanical phytochemical combination PB125 protects endothelial cells from oxidative stress-induced injury [21-23] suggesting the potential vascular benefits may be greater with aging or in more pronounced states characterized by heightened oxidative stress or impaired antioxidant signaling. Therefore, transient Nrf2 activation with PB125 may help mitigate age- and disuse-related vascular dysfunction. However, the effects of PB125 on age- and disuse-related vascular function in younger and older adults remain unknown.

The purpose of this study was to determine 1) the effects of PB125 on vascular function in younger and older adults, and 2) whether PB125 attenuates disuse-induced vascular dysfunction following bed rest in older adults and limb immobilization in younger adults. We hypothesized that PB125 would improve vascular function in older, but not younger adults, and prevent vascular dysfunction following bed rest and limb immobilization.

## 2. Methods

### Ethical approval

All experimental study protocols and procedures were approved by the Institutional Review Board at the University of Utah (IRB #111321) and the Salt Lake City Veterans Affairs Medical Center (VAMC). The nature, benefits and risks associated with this study were explained to all participants before written and verbal consent was obtained prior to participation. This study conformed to the standards set by the *Declaration of Helsinki*.

### 2.1. Study design, randomization, supplementation and disuse

This study was a randomized, double-blind, placebo-controlled study design. All participants consumed either two capsules (100 mg/capsule) of PB125 (Pathways Bioscience, Aurora, CO) or placebo (cellulose) totaling 200 mg/day for 2 weeks (Fig. 1A). Older participants continued supplementation for 5 days during bed rest (acute hospitalization) whereas younger participants continued supplementation for 2 weeks during limb immobilization (knee brace and crutches) as two separate experimental models of disuse (Fig. 1B). The bed rest protocol consisted of a 5-day hospital stay at the University of Utah Hospital. Participants remained in bed for the duration of the intervention and were monitored 24 h/day by the hospital nursing staff to ensure protocol adherence and participant safety. All activities of daily living were performed with the assistance of the nursing staff to prevent unintended physical activity/weight bearing activities. The limb immobilization protocol consisted of using a knee brace fixed at 60 degrees of knee flexion to restrict knee joint movement and was worn on the left leg for standardization across all participants and to allow participants to safely perform activities of daily living, particularly driving. Participants were instructed to use crutches for activities of daily living to minimize weight bearing activity on the immobilized leg while maintaining normal activity on the opposite leg. The removal of the knee brace was permitted for hygiene related purposes.

PB125 is a nutraceutical compound composed of rosemary (*rosmarinus officinalis*), ashwagandha (*withania somnifera*), and luteolin extracts containing the active ingredients of carnosol, withaferin A and luteolin. Participants were instructed to consume one capsule in the morning and one capsule at night of PB125 or placebo with or after food at the same time of day. Compliance with the supplementation protocol was determined through evaluation of circulating plasma carnosic acid concentration. Participants were block randomized by an individual that was not involved in experimental implementation or data analyses. PB125 and placebo capsules were distributed by the Salt Lake City VAMC Investigational Pharmacy and encapsulated in a manner to prevent visual identification of the supplement.

These studies involved three main visits. Visit 1 (~3 h) served as the baseline visit consisting of resting measures of heart rate (HR) and blood pressure (BP) followed by assessments of microvascular function (passive leg movement, [PLM]), macrovascular function (brachial and popliteal flow-mediated dilation, [FMD]), and blood biomarkers. All assessments were repeated on visit 2 (~3 h) which served as the post supplementation visit and visit 3 (~3 h) which served as the post visit for either limb immobilization for young or bed rest for old, respectively.

### 2.2. Participants

Twenty younger adults (8M/12F, mean age 27 ± 7 y) and twenty-four older adults (12M/12F, mean age 67 ± 8 y) were randomized to receive either placebo or PB125. All younger participants were free of overt CVD and not prescribed any medications. Older participants were excluded if they presented with a history of cardiovascular or endocrine disease, cancer, liver or respiratory disease, stroke with motor disability, risk of deep vein thrombosis, use of blood thinners, currently on a weight-loss diet, or had uncontrolled hypertension and/or implanted electronic devices. All participants reported to the Utah Vascular Research Laboratory (UVRL) located in the Salt Lake City VAMC Geriatric, Research, Education and Clinical Center for all study protocols after an overnight fast (8-12 h) from food, caffeine, alcohol and exercise as previously described [24].

### 2.3. Anthropometrics, resting heart rate, blood pressure, and physical activity

Upon arrival at the laboratory, anthropometric measurements of height and weight were used to calculate body mass index (BMI). Height and weight were measured to the nearest 0.1 cm and 0.1 kg using a stadiometer and electronic scale. Resting HR was assessed by a 3-lead electrocardiogram (AcqKnowledge, BIOPAC systems) while resting BP was measured using an automated sphygmomanometer (Tango M2, SunTech Medical) following 5 min of uninterrupted sitting in a quiet, thermoneutral room. All measures were assessed in triplicate and averaged for all analyses. All participants also wore an accelerometer (GT1M: Actigraph, Pensacola, FL, USA) for a minimum of fourteen days to assess baseline physical activity status (average steps/day). Younger participants continued to wear the accelerometer for the entirety of the limb immobilization protocol to ensure physical activity was reduced during the experimental protocol. Physical activity was not assessed during bed rest in older adults since all study participants were monitored by the hospital nursing staff and no ambulation was allowed.

### 2.4. Blood chemistry and antioxidant status

Venous blood samples were obtained from the antecubital vein at baseline, 2 weeks post supplementation and following the disuse protocols in the fasted condition. Standard blood chemistry including total cholesterol, triglycerides, low-density lipoprotein cholesterol (LDL-C), high-density lipoprotein cholesterol (HDL-C), blood glucose, hematocrit and hemoglobin concentration were analyzed by the Veterans Affairs blood laboratory. Blood samples were stored at −80 °C for future analysis. Serum antioxidant status was assessed by superoxide dismutase (SOD, Invitrogen, Cat No. EIASODC) in duplicate using an enzyme-linked immunoassay.

### 2.5. Carnosic acid metabolite detection

In a subsample of participants (n = 19, 10* older adults, and 9* younger adults) plasma was used to determine carnosic acid to confirm both compliance and systemic exposure to the active ingredient of PB125. PB125 increased circulating plasma levels of carnosic acid (baseline: 1998 ± 1999 vs post disuse: 179,297 ± 184,553, peak integration AUC, p < 0.0001) compared to placebo (baseline: 6137 ± 8603 vs post disuse: 3342 ± 4,023, peak integration AUC, p = 0.22) confirming both compliance and systemic exposure to the active ingredient of PB125.

### 2.6. Extraction method

Forty microliters of plasma were combined with 160 μL of ice-cold acetonitrile (ACN): methanol containing 0.1% formic acid and 1 μg/mL of anthracene-9-carboxylic acid (9AC) internal standard. A process blank was created at this time containing only extraction solvent and 40 μL of ddH2O and carried through the extraction process. All samples were vortexed for 30 s prior to incubation at −20 °C for 1 h. The samples were then centrifuged for 10 min at 20,000 rcf and 4 °C. The supernatant was transferred to PTFE autosampler vials for analysis. A quality control sample was created by pooling equal amounts from each sample of its respective type. The samples were randomized prior to analysis, and the QCs analyzed every 4-5 samples.

### 2.7. LC/MS method

Ultra performance liquid chromatography-Mass Spectrometry was performed using a SCIEX Nexera coupled to a SCIEX 6500 Q-Trap mass spectrometer in negative-ionization mode. Separation was achieved using a Waters Aquity CSH C18 100 × 2.1 mm column with a gradient elution of 0.1 % formic acid in water (eluent A) and 0.1% formic acid in ACN at a flow rate of 0.5 mL/min. Starting conditions of 5% eluent B were held for 0.5 min, then increased linearly to 99% eluent B over 9.5 min. Eluents were returned to starting conditions over 0.1 min, and the system was allowed to equilibrate for 3 min between runs. Transitions for carnosic acid (331/287, 331/271, 331/215), and 9AC (221/177) were monitored by multiple reaction monitoring. Source conditions were optimized for carnosic acid and were Curtain Gas = 20 L/min, Collision Gas = 12, IonSpray Voltage = −4000 V, Temperature = 500 °C, Ion Source Gas 1 = 20 L/min, Ion Source Gas 2 = 40 L/min. Data were analyzed by SCIEX MultiQuant 3.0.3. Areas reported were normalized to 9AC.

### 2.8. Passive leg movement

Passive leg movement (PLM) was performed as previously described [25]. Using doppler ultrasonography (Logiq E9, GE Medical Systems, Milwaukee, WI) local measures of common femoral artery diameter and velocity were assessed longitudinally 2-3 cm proximal to the bifurcation using a 9-14 MHz linear array probe. In brief, participants were seated in the upright position with their legs fully extended (180°) while baseline measures of blood velocity and central hemodynamics were recorded for 60 s. Following baseline measures, a member of the research team controlled the movement of the experimental lower leg back and forth (i.e., passive knee flexion and extension) through ~90° range of motion at a cadence of 1 Hz (metronome guided) for 60 s. Passive leg movement induced blood velocity and central hemodynamics were recorded for 60 s following the onset of passive knee-flexion extension. Replicate (2) PLM tests were separated by ~5 min of quiet recovery. Leg blood flow (LBF) was calculated as: LBF (mL * min^−1^) = [60 x V_mean_ x *π* (vessel diameter/2)^2^] and leg vascular conductance (LVC) was calculated as: LVC (mL•min•mmHg^−1^) = LBF/mean arterial pressure (MAP). Peak changes in LBF (Δ LBF_peak_) and LVC (Δ LVC_peak_) were calculated as peak minus baseline values. The area under the curve (AUC) for LBF (LBF_AUC_) and LVC (LVC_AUC_) were calculated as the summed second-by-second response above baseline during the 60 s of PLM as previously described [26]. Heart rate was monitored using a 3-lead electrocardiogram (BioPac) and beat-by-beat changes in central hemodynamics including cardiac output (CO), stroke volume (SV), and MAP were determined via finger photoplethysmography (Finometer: Finapress Medical Systems, Amsterdam, the Netherlands). Finometer derived measurements of MAP were normalized to MAP determined from the use of an automated sphygmomanometer (Tango^+^, Suntech, Morrisville, NC). MAP was calculated as diastolic blood pressure +1/3 (systolic blood pressure – diastolic blood pressure).

### 2.9. Brachial and popliteal artery flow-mediated dilation

Flow-mediated dilation (FMD) testing was performed in accordance with established guidelines [27]. All participants rested in the supine position for ~10 min prior to testing. Brachial artery FMD was performed first followed by popliteal artery FMD. Popliteal artery FMD was performed on the immobilized leg in young adults across all three visits. The rapid inflation cuff was placed around the participants’ forearm immediately distal of the antecubital fossa for brachial artery FMD and on the upper calf, distal to the knee and distal to the doppler probe measurement site for popliteal artery FMD. Following ~1 min of baseline measures of brachial and/or popliteal artery diameter and velocity the forearm and/or calf blood pressure cuffs were inflated to 250 mmHg for 5 min and then rapidly deflated while data were acquired over a 2 min period to obtain the peak vasodilatory response. Brachial artery and popliteal artery vasodilation was determined offline from end-diastolic, ECG R-wave triggered images collected from the ultra-sound using an automated edge detection software (Brachial Analyzer, medical Imaging Applications). FMD was quantified as the maximal change in artery diameter after cuff release, expressed as a percentage increase from baseline diameter (% FMD). Shear rate was calculated as: SR (s^−1^) = blood velocity*8/vessel diameter. Reactive hyperemia was calculated as the summed blood flow response over time after normalizing for baseline blood flow. Coefficient of variance for FMD assessments in a subgroup of individuals was 5.12 ± 0.72%.

### 2.10. Statistical analysis

Sample size was determined *a priori* using G*Power and indicated that n = 10 per group would provide α = 0.05 (two-tailed) and 1–β = 0.80 to detect a 2.0 ± 2.0% decrease in FMD with bed rest/immobilization or a 1.0 ± 1.0% increase with PB125. All statistical analyses were performed using GraphPad prism (Version 10.6.1). An independent (unpaired) two-tailed samples *t*-test was used to compare baseline characteristics between PB125 and placebo groups for younger and older adults. No comparisons were made between younger and older adults at baseline, in response to PB125, or in response to disuse. The effects of PB125 and placebo on brachial and popliteal FMD, PLM-induced changes in LBF and LVC, and serum SOD concentrations were analyzed using a mixed-effects two-way ANOVA with a Fisher LSD post hoc analysis for time X supplement. A Fisher LSD post hoc analysis was used given the pilot, and hypothesis driven nature of this study, where minimizing type II error was a priority. For aim 1, time refers to baseline and 2 weeks of supplementation and supplement refers to PB125 or placebo. For aim 2, time refers to 2 weeks of supplementation and either 2 weeks of limb immobilization (young) or 5 days of bed rest (old) whereas supplement refers to PB125 or placebo. An independent (unpaired) two-tailed samples *t*-test was used to compare differences between the relative changes for serum SOD concentrations with PB125 and placebo. A p < 0.05 was deemed statistically significant for all analyses. Data are mean ± standard deviation.

## 3. Results

A total of twenty younger adults (8M/12F, mean age 27 ± 7 y) completed the limb immobilization protocol and twenty-four older adults (12M/12F, mean age 67 ± 8 y) completed the bed rest protocol. Baseline characteristics and comorbid conditions of the participant populations for younger and older adults are presented in Tables 1 and 2. No differences in participant characteristics were observed in the placebo or PB125 group for either young or older adults. All young participants in the limb immobilization protocol decreased physical activity by ~44 % (~4000 steps) in the placebo group and ~56% (~3600 steps) in the PB125 group (independent samples *t-test*, absolute change, p = 0.731).

### 3.1. PB125 improves vascular endothelial function in older adults

Two weeks of supplementation with PB125 in older adults increased brachial artery FMD by +65% (PB125: 2.8 ± 1.5 to 3.8 ± 1.3%, p < 0.0001, Fig. 2A) and popliteal artery FMD by +57% (PB125: 1.5 ± 1.5 to 2.3 ± 1.4%, p = 0.033, Fig. 2B). No changes were observed in younger adults for either brachial artery FMD (PB125: 6.2 ± 2.7 to 5.0 ± 2.0%, p = 0.064, Fig. 2C) or popliteal artery FMD (PB125: 3.4 ± 2.6 to 4.4 ± 2.8%, p = 0.271, Fig. 2D). Two weeks of supplementation did not improve PLM-induced leg blood flow or leg vascular conductance in either age group (all, p > 0.05, Fig. 3A-D).

### 3.2. PB125 prevents disuse-induced vascular dysfunction in older adults following bed rest

Following 1 week of bed rest in older adults, brachial artery FMD decreased by ~46% (3.8 ± 1.3 to 1.9 ± 0.9%, p < 0.0001, Fig. 4A) and popliteal artery FMD decreased by ~64% (1.4 ± 1.1 to 0.6 ± 0.8%, p = 0.015, Fig. 4B) which was preserved in older adults consuming PB125 (brachial artery FMD: 3.8 ± 1.3 to 3.6 ± 1.4%, p = 0.543, Fig. 4A, and popliteal artery FMD: 2.3 ± 1.4 to 2.7 ± 1.4%, p = 0.269, Fig. 4B).

Similarly, the PLM-induced change in peak leg blood flow (ΔLBF_PEAK_: 422 ± 171 to 353 ± 127 mL•min^−1^, p = 0.025, Table 5) and overall vasodilatory response to PLM, assessed by leg vascular conductance AUC (LVC_AUC_: 1.6 ± 1.1 to 1.1 ± 0.7, p = 0.049, Fig. 4D) were also decreased during 5 days of bed rest which was fully preserved in older adults consuming PB125 (all, p > 0.05, Table 5 and Fig. 4C-D). FMD characteristics, PLM-derived central hemodynamics, diameter and resting blood flow and conductance in older adults are presented in Tables 3 and 7.

### 3.3. PB125 prevents lower limb vascular dysfunction in younger adults following limb immobilization

Following 2 weeks of limb immobilization in young adults, popliteal artery FMD decreased by ~50% (4.8 ± 2.9 to 2.4 ± 1.7%, p = 0.032, Fig. 5B) and was preserved in younger adults consuming PB125 (4.4 ± 2.8 to 3.3 ± 1.8%, p = 0.302, Fig. 5B). Brachial artery FMD was unchanged during limb immobilization (placebo: 5.8 ± 1.9 to 6.1 ± 1.8%, p = 0.698 vs PB125: 5.0 ± 2.0 to 5.3 ± 3.1%, p = 0.722, Fig. 5A). No changes were observed in any of the PLM derived outcomes (ΔLBF_PEAK_, ΔLBF_AUC_, ΔLVC_PEAK_, ΔLVC_AUC_) following limb immobilization in young adults (all, p > 0.05, Table 6 and Fig. 5C-D). Flow-mediated dilation characteristics, PLM-derived central hemodynamics, diameter and resting blood flow and conductance in younger adults are presented in Tables 4 and 8.

### 3.4. PB125 preserves antioxidant capacity during disuse

Serum antioxidant capacity, assessed by circulating SOD concentrations decreased (31.8 ± 10.0 to 28.3 ± 7.2 U/mL, interaction, p = 0.021, Fig. 6A) following 2 weeks of supplementation with PB125 in older adults. Serum antioxidant capacity, assessed by circulating SOD concentrations decreased (30.6 ± 8.3 to 25.3 ± 4.2 U/mL, p = 0.015, Fig. 6B) following 5 days of bed rest which was preserved in older adults consuming PB125 (28.3 ± 7.2 to 28.6 ± 8.6 U/mL, p = 0.888, Fig. 6B). The relative change (−14%, p = 0.091, data not shown) in serum SOD concentrations was not statistically different between placebo and PB125 during bed rest in older adults. PB125 did not increase SOD concentrations with 2 weeks of supplementation nor decrease during limb immobilization in younger adults (both, p > 0.05, Fig. 7A-B). Yet, the relative change in SOD concentrations tended to decrease (−8%, p = 0.051, data not shown) in the placebo group during limb immobilization in young adults.

## 4. Discussion

This study provides novel evidence that 1) short-term supplementation (2 weeks) with PB125 improves brachial and popliteal artery FMD in older, but not younger adults, 2) PB125 preserves brachial and popliteal artery FMD, microvascular vasodilation capacity measured by leg vascular conductance during PLM, and circulating antioxidant capacity following bed rest in older adults, and 3) PB125 preserves popliteal artery FMD following limb immobilization with a tendency for preserved antioxidant capacity in younger adults. These results advance prior studies demonstrating that PB125 activates Nrf2 [23] and suggest that the beneficial effects of PB125 on age- and disuse-related vascular function are attributable, in part, to enhanced Nrf2 signaling and Nrf2-related antioxidant defense mechanisms. Collectively, these findings provide clinically relevant evidence for targeted Nrf2 activation via PB125 as a novel nutraceutical to improve vascular function in older adults and mitigate vascular dysfunction during periods of physical inactivity.

### 4.1. Effects of PB125 on age-related vascular function

Age-related vascular endothelial dysfunction, assessed by brachial artery FMD, is a strong independent predictor of increased CVD risk [28]. To the best of our knowledge, this study represents the first human-based investigation using targeted Nrf2 activation to improve age-related vascular dysfunction. Herein, we demonstrate for the first time a PB125-enhancing effect on brachial artery FMD by +65 % (+1% absolute) and popliteal artery FMD +57% (+0.84% absolute) in older adults. This is noteworthy as similar improvements in FMD (+1-2.5%, absolute) have been observed in middle-aged and older adults over long-term nutraceutical interventions [29-32]. Our findings support and extend these findings demonstrating PB125 elicits comparable improvements in FMD, despite a shorter intervention period, highlighting the efficacy of PB125 relative to that of other nutraceutical compounds in older adults. However, it is important to consider that PB125 improved FMD to levels comparable to the placebo condition, suggesting that the observed improvement may reflect regression toward the mean rather than a fully independent treatment effect. Given the inherent variability with FMD, coupled with the modest sample size of this pilot study, larger and more adequately powered studies are needed to confirm the magnitude of the PB125-mediated improvements in vascular function.

Mechanistically, PB125 activates Nrf2 signaling, increasing antioxidant gene expression, and improving redox balance [23]. Carnosic acid and its metabolite, carnosol, activate the Nrf2 pathway via the dissociation of Keap1 from Nrf2 thus allowing it to translocate to the nucleus and bind to the antioxidant response element [23]. Current preclinical evidence demonstrates that its bioactive compounds improve endothelial cell resiliency, enhance NO production, and protect against oxidative stress, supporting a plausible mechanism for improved vascular function [33,34]. Given that aging and physical inactivity are associated with dysregulated Nrf2 signaling and altered redox balance [35,36], the PB125-related improvements and preservation of vascular function during disuse likely reflects augmented antioxidant signaling in the presence of oxidative stress.

Conversely, PB125 did not exert an effect on brachial or popliteal artery FMD in younger adults likely reflecting favorable endothelial function, enhanced NO bioavailability, and redox balance that limit the capacity for improvement [1,37,38]. Greater or prolonged Nrf2 activation in young adults may blunt ROS-mediated signaling mechanisms that are important for vascular function [39] as evidenced by a down-ward trend toward reduced brachial artery FMD in young adults following 2 weeks of supplementation. PB125 also did not improve PLM-induced leg blood flow or leg vascular conductance following short-term supplementation with PB125 in either age group. In older adults, the PB125-related improvements in FMD without a concomitant improvement in leg blood flow or leg vascular conductance suggests that PB125 preferentially enhances endothelial NO signaling in conduit arteries, without sufficiently modulating resistance artery vasodilator function or microvascular control which may rely more on NO-independent signaling mechanisms with aging [40-42]. Overall, these findings demonstrate that PB125 confers clinically meaningful improvements in conduit artery endothelial function in older adults while highlighting limited redox plasticity within the aged microvasculature that restricts improvements in leg blood flow and vascular conductance.

### 4.2. Effects of PB125 on disuse-related vascular dysfunction in older adults

Vascular dysfunction associated with physical inactivity and aging appears to share similar mechanisms including increased oxidative stress and impaired NO bioavailability. Experimental models of disuse, including bed rest and limb immobilization have been extensively used as methods to mechanistically interrogate inactivity-induced vascular dysfunction and target these interrelated pathways.

Short-term (5-10 days) bed rest induces vascular dysfunction including decreased brachial artery FMD and leg vascular conductance [43-46]. Yet, these findings are primarily limited to younger and/or mixed-aged cohorts with little to no evidence for the effects of short-term bed rest in older adults exclusively, who are at an increased risk of CVD. To the best of our knowledge, this study is one of the first to demonstrate substantial decreases in both macro- and microvascular function evoked by 5 days of bed rest in older adults. Specifically, our data demonstrate brachial artery FMD and popliteal artery FMD decreased by ~47% (−1.85%, absolute) and ~64% (−0.81%, absolute), respectively, which is comparable to that of young adults (~2-2.5% absolute) [43]. Additionally, we observed a ~32% reduction in PLM-induced leg vascular conductance in older adults, which is consistent with previous reports in young adults (~30-35%) following 10 days of bed rest [44]. More importantly, these declines in macro- and microvascular function were not observed with PB125 supplementation, indicating preservation of vascular function during short-term disuse in older adults. Consistent with this, PB125 preserved circulating SOD concentrations suggesting that PB125 maintains NO bioavailability and redox status during bed rest which may underlie the potential protective effects of PB125 on vascular function during disuse. These findings indicate short-term bed rest impairs vascular function in older adults, and that interventions targeting Nrf2 activation effectively preserve vascular function during extended periods of disuse with aging.

### 4.3. Effects of PB125 on disuse-related vascular dysfunction in young adults

In young adults, limb immobilization provides a robust approach to induce vascular dysfunction [2,47,48]. Our data demonstrates that 14 days of limb immobilization resulted in a ~26% decrease in popliteal (lower limb) artery FMD (−2.4 % absolute), despite a reduction in popliteal artery diameter and increase in shear stress. This decrease in arterial diameter coupled with increased shear stress may contribute to a greater impairment in FMD as the endothelium, although exposed to a greater shear stimulus, likely experiences decreased NO bioavailability and potentially greater oxidative stress, limiting vasodilatory capacity. Compared to previous work, these changes occurred without a corresponding decrease in PLM-induced leg blood flow or vascular conductance following limb immobilization suggesting that microvascular function may be preferentially preserved during limb immobilization in young healthy adults compared to lower limb conduit artery macrovascular function [2,49]. Importantly, this decrease in popliteal artery FMD with limb immobilization was not observed in the PB125 condition, indicating that PB125 preserved lower limb conduit artery endothelial function during limb immobilization. These data suggest that lower limb conduit artery vascular function is more sensitive to changes during limb immobilization and that targeted Nrf2 activation may offer an effective therapeutic approach to maintain macrovascular endothelial function during inactivity in young adults.

Age- and disuse-induced vascular dysfunction during limb immobilization and bed rest results from altered hemodynamics, reduced NO bioavailability and increased oxidative stress, which collectively impair endothelial function [43,46,50,51]. Aging amplifies these maladaptation's as older adults typically exhibit higher oxidative stress, reduced antioxidant defenses, impaired endothelial NO synthase (eNOS) activity and increased arterial stiffness [1,8,52]. Consequently, the endothelium's diminished adaptability during disuse promotes eNOS uncoupling, excessive ROS production, and macro- and microvascular remodeling leading to impaired arterial compliance and vascular dysfunction.

Baseline physical activity is also a potent modulator of vascular function as higher levels of physical activity confers a vascular protective effect associated with higher endothelial function and antioxidant capacity and lower oxidative stress compared to inactive adults [53]. Interestingly, higher baseline levels of physical activity (average steps/day) tended *(r* = *−0.61, p*=*0.06, data not shown)* to be associated with a greater reduction in popliteal artery FMD following limb immobilization in young adults. These data suggest that individuals with higher habitual physical activity levels have greater vascular plasticity, potentially leading to a greater relative decline in vascular function during periods of disuse.

### 4.4. Strengths and limitations

The primary strength of this study was the double-blind, randomized, placebo-controlled study design to demonstrate a PB125-enhancing effect of vascular function in older adults and preserved vascular function following two separate experimental models of disuse. Of note, this study is not without limitations. First, the disuse protocols in older (bed rest) and younger (unilateral limb immobilization) adults were intentionally different, thereby limiting age-related comparisons during disuse. The rationale for using different disuse protocols was based on safety, feasibility, and clinical relevance. Short-term bed rest in older adults closely mimics acute hospitalization which enhances translational applicability to aging and disease populations [7]. Conversely, limb immobilization in younger adults provides a well-established, localized model of disuse that reliably induces vascular dysfunction with minimal risk [2,47]. Differences in the duration of disuse (5 days vs. 14 days), combined with systemic effects of bed rest versus localized effects of limb immobilization, influenced the magnitude and mechanisms associated with vascular dysfunction observed in each age group. Indeed, bed rest induces systemic hemodynamic alterations and autonomic dysfunction, and reductions in shear stress, whereas limb immobilization produces primarily local reductions in blood flow and shear stress within the immobilized limb. Therefore, direct comparisons between age groups were avoided, as the observed differences in vascular function may reflect modality-specific vascular responses in addition to age-related physiological differences. Future studies are warranted to isolate the independent effects of aging more directly versus disuse modality on vascular dysfunction. Second, the current sample, albeit well-matched for sex and baseline characteristics, consists of a primarily homogenous sample of Caucasian adults potentially limiting the generalizability of these findings. Third, sex hormones were not assessed in younger or older adult males and females limiting the interpretation for the role of testosterone and estrogen on vascular function with disuse. Fourth, we did not directly assess measures of oxidative stress or Nrf2 signaling, however, we provide evidence for PB125 to preserve antioxidant capacity following disuse with support from previous findings demonstrating a protective role of PB125 on endothelial cell biology in the presence of oxidative stress and its ability to activate Nrf2 under oxidative stress conditions. Lastly, our study involved a modest sample size which may have limited statistical power to detect small changes in our primary vascular outcomes. However, our changes in both FMD and PLM were consistent with prior literature following bed rest and limb immobilization and resulted in clinically meaningful improvements. Nevertheless, future investigations using a larger and more diverse population to examine the long-term and dose-response effects, pharmacokinetics and potential impact of sex and hormone-specific effects of PB125 during periods of disuse in younger and older adults are needed to extend the current findings.

## 5. Conclusion

These findings demonstrate targeted Nrf2 activation with PB125 improves age-related vascular endothelial dysfunction while preserving vascular function and antioxidant capacity following periods of disuse in younger and older adults. Collectively, these data support PB125 as a novel therapeutic strategy to improve and preserve vascular function in humans with aging and disuse and potentially reduce the risk of future CVD.

## Figures and Tables

**Fig. 1.**
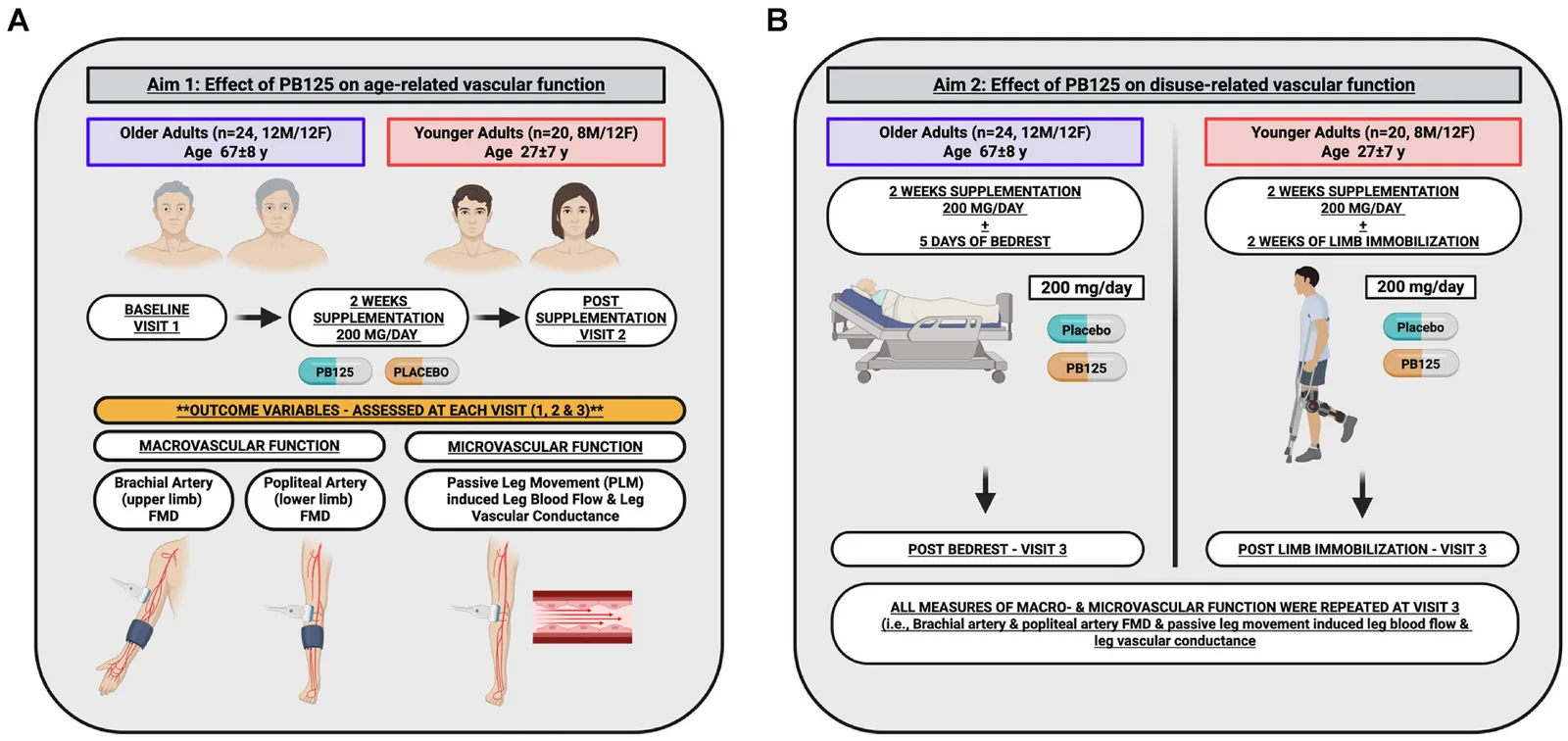
Study design schematic depicting A) the effect of short-term supplementation with PB125 (200 mg/day) on vascular function in older and younger adults and B) the effect of PB125 on vascular function following 5 days of bedrest in older adults and 2 weeks of limb immobilization in young adults.

**Fig. 2.**
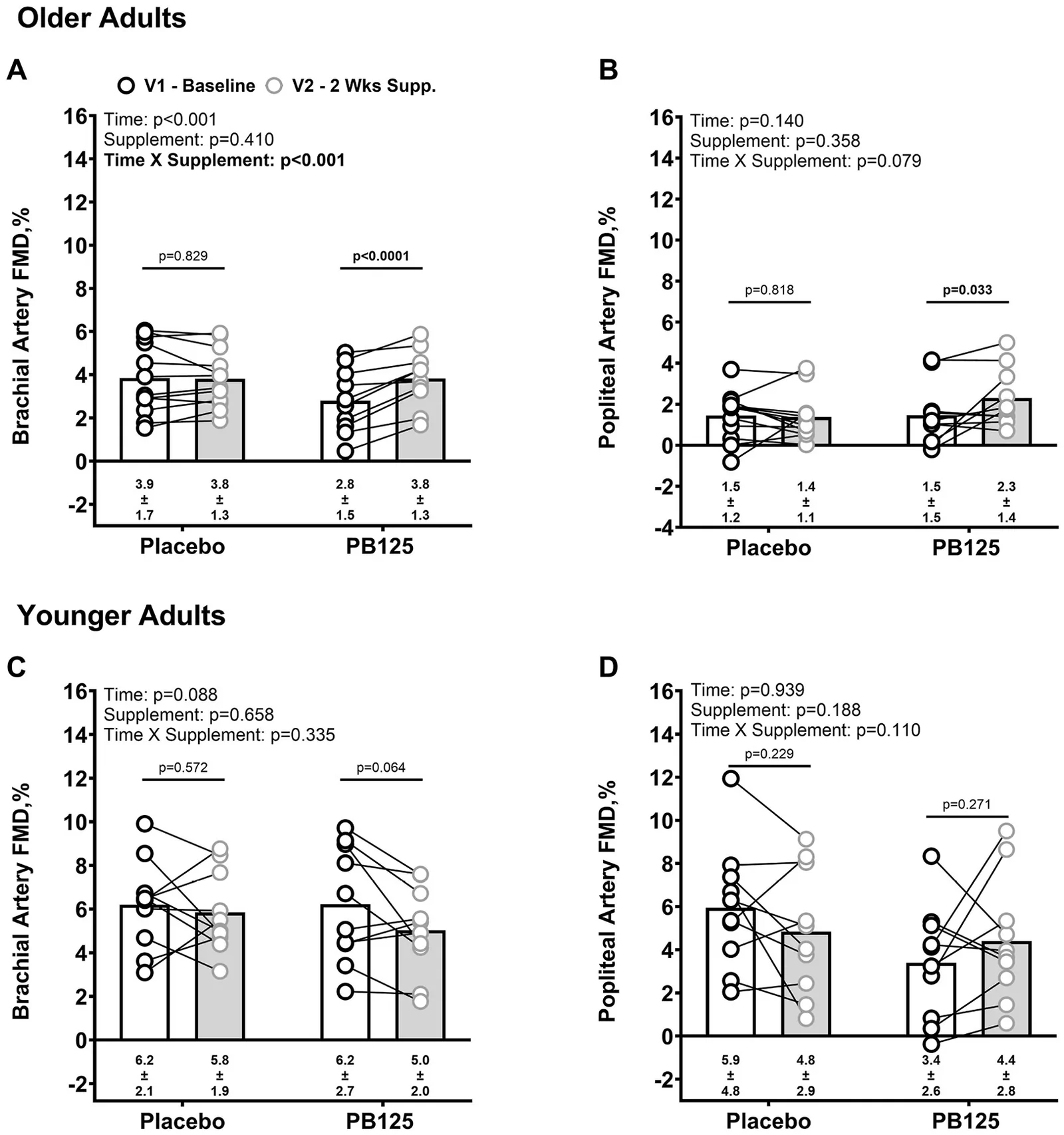
Effect of PB125 on brachial and popliteal artery FMD in younger and older adults. A) brachial artery FMD expressed as a percentage in older adults, B) popliteal artery FMD expressed as a percentage in older adults, C) brachial artery FMD expressed as a percentage in young adults, and D) popliteal artery FMD expressed as a percentage in young adults. FMD, flow-mediated dilation; 2 Wks. Supp. 2 weeks of supplementation. Data are mean ± SD. Old Placebo n = 12, Old PB125 n = 10, Young Placebo, n = 10, Young PB125 n = 10.

**Fig. 3.**
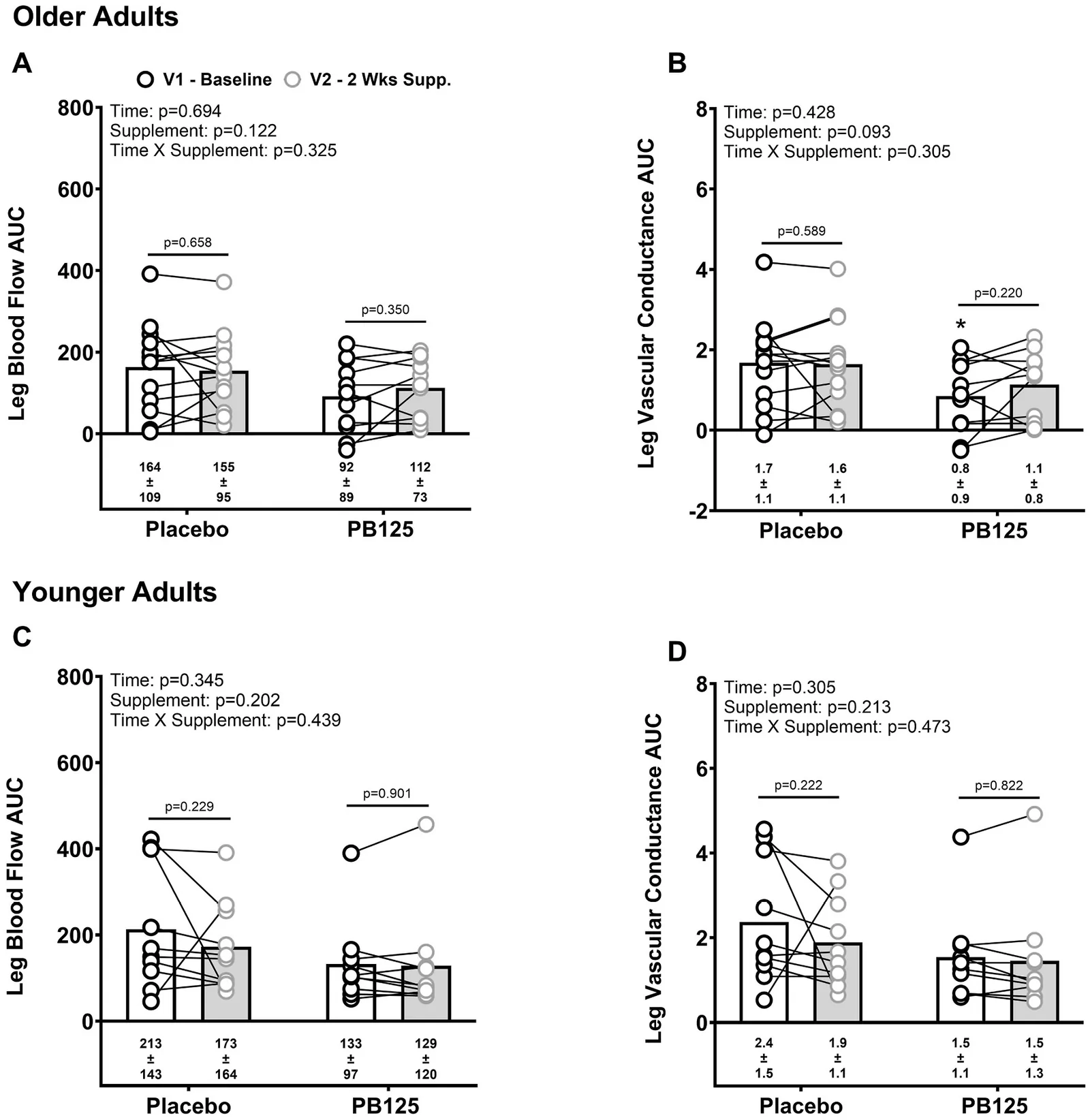
Effect of PB125 on PLM-induced leg blood flow and leg vascular conductance in younger and older adults. A) leg blood flow AUC in older adults, B) leg vascular conductance AUC in older adults, C) leg blood flow AUC in young adults, and D) leg vascular conductance AUC in young adults. PLM, passive leg movement; 2 Wks. Supp. 2 weeks of supplementation; AUC, area under the curve. Data are mean ± SD. Old Placebo n = 13, Old PB125 n = 11, Young Placebo, n = 10, Young PB125 n = 10.*p < 0.05 vs baseline placebo.

**Fig. 4.**
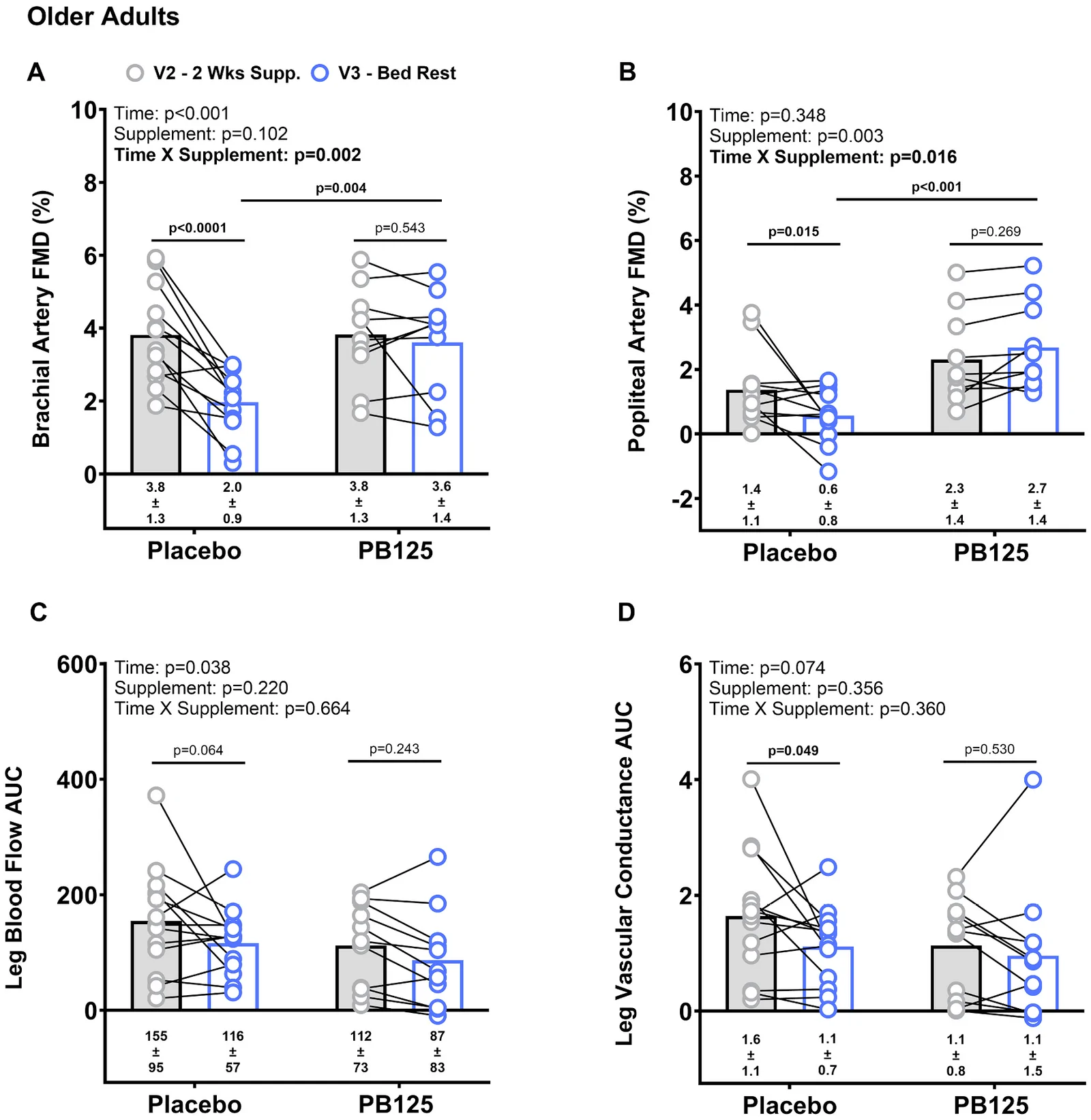
Effect of PB125 on vascular function in older adults following bed rest. A) brachial artery FMD expressed as a percentage in older adults, B) popliteal artery FMD expressed as a percentage in older adults, C) leg blood flow AUC in older adults and D) leg vascular conductance AUC in older adults. FMD, flow-mediated dilation; 2 Wks. Supp. 2 weeks of supplementation; AUC, area under the curve. Data are mean ± SD. Brachial and popliteal artery FMD: Placebo n = 12, PB125 n = 10, Leg blood flow and vascular conductance: Placebo n = 13, PB125 n = 11.

**Fig. 5.**
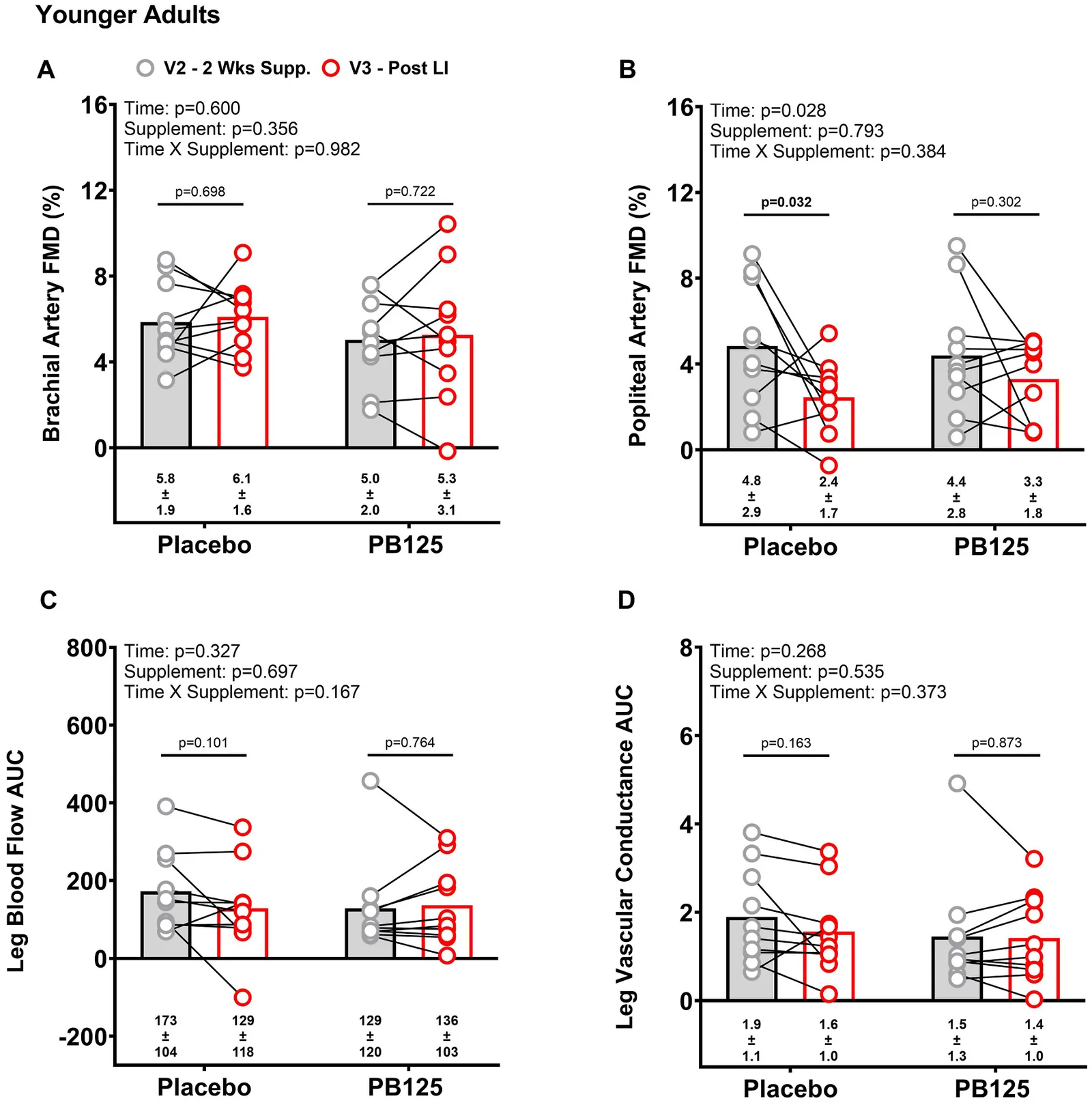
Effect of PB125 on vascular function in young adults following limb immobilization. A) brachial artery FMD expressed as a percentage in young adults, B) popliteal artery FMD expressed as a percentage in young adults, C) leg blood flow AUC in young adults and D) leg vascular conductance AUC in young adults. FMD, flow-mediated dilation; 2 Wks. Supp. 2 weeks of supplementation; AUC, area under the curve. Data are mean ± SD. Placebo, n = 10, PB125 n = 10.

**Fig. 6.**
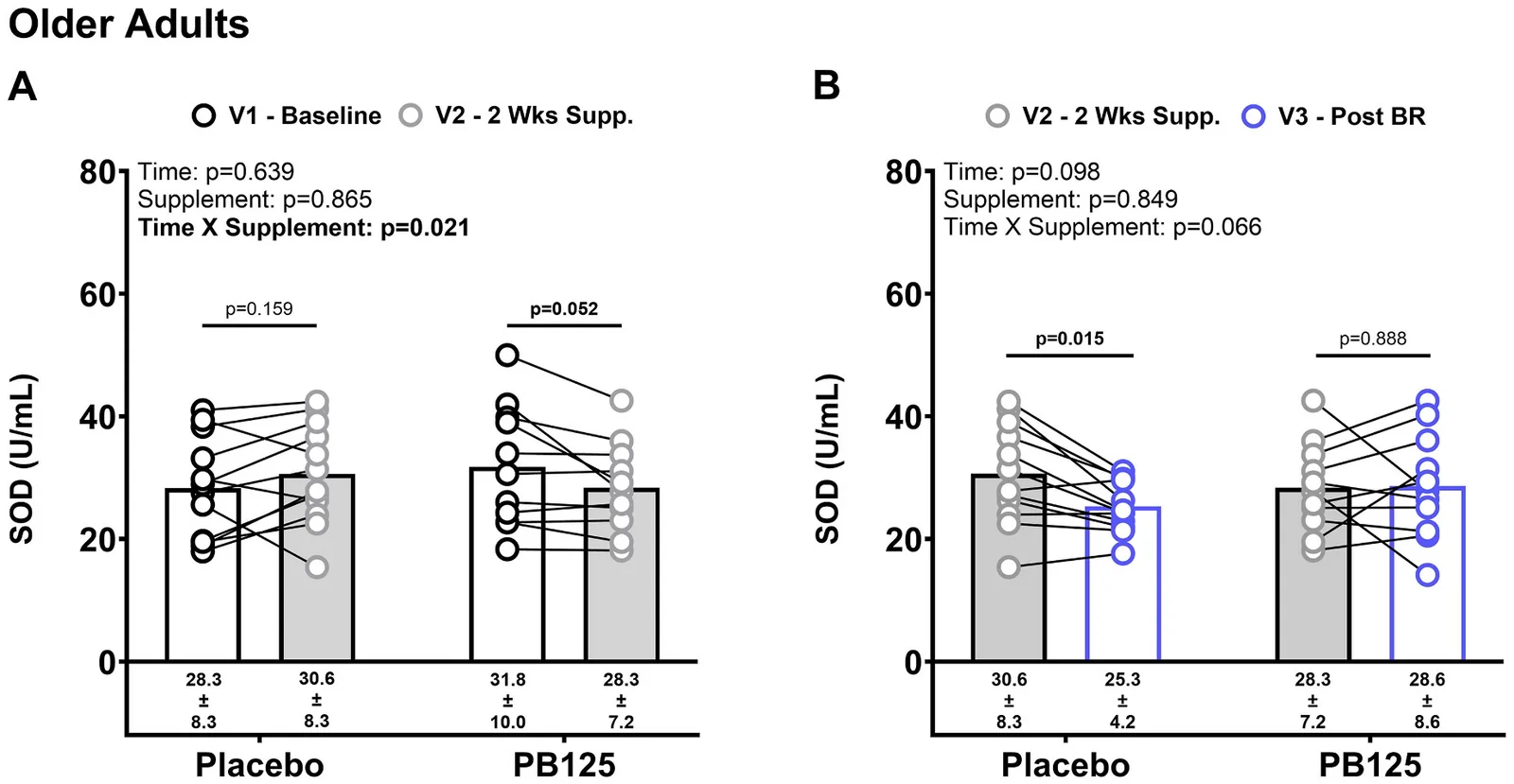
Effect of PB125 on antioxidant capacity in older adults. A) serum SOD concentrations following 2 weeks of PB125 or placebo supplementation in older adults and B) serum SOD concentrations following 1 week of bed rest in older adults. SOD, superoxide dismutase; 2 Wks. Supp., 2 weeks supplementation. Data are mean ± SD. Placebo n = 12, PB125 n = 11.

**Fig. 7.**
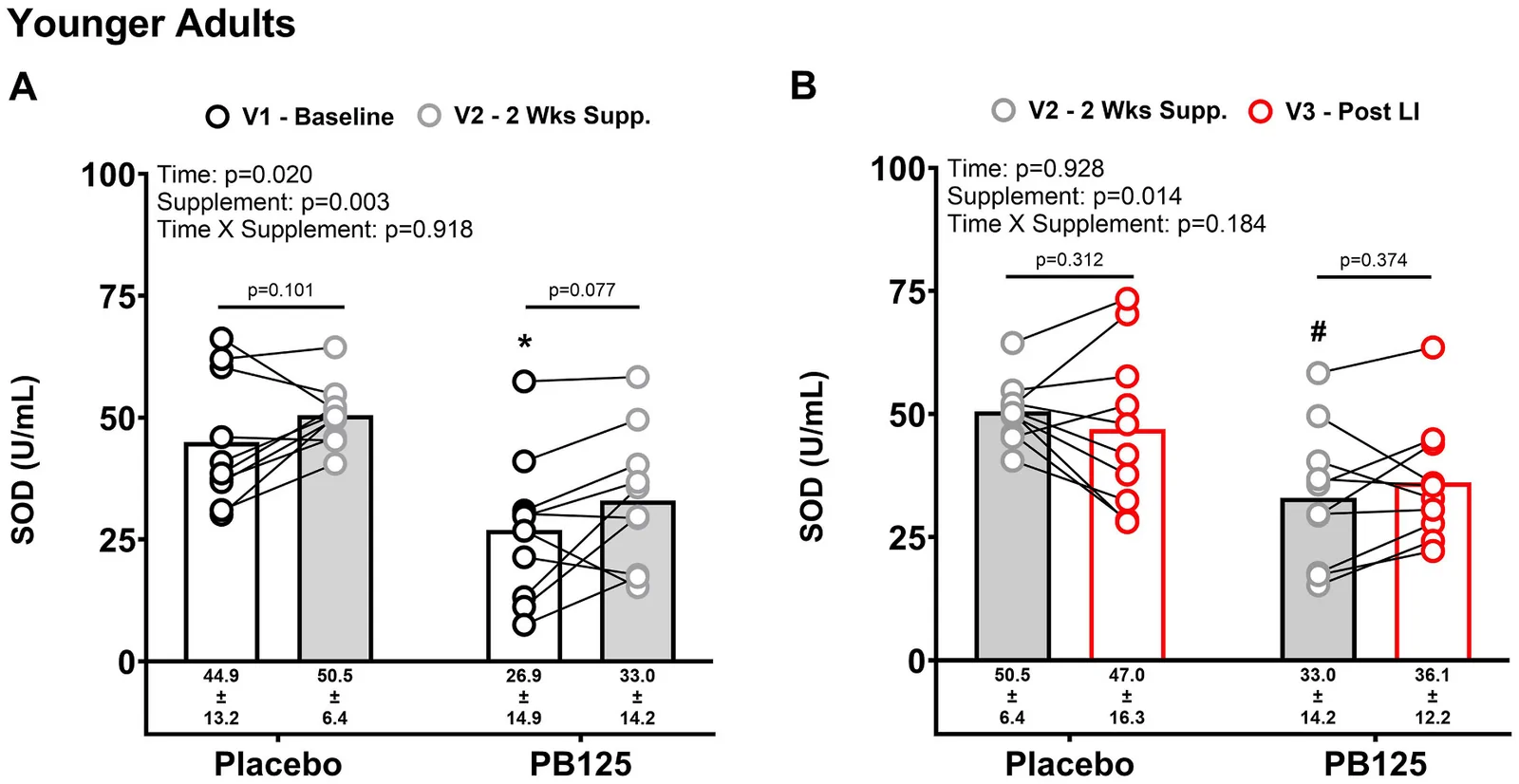
Effect of PB125 on antioxidant capacity in young adults. A) serum SOD concentrations following 2 weeks of PB125 or placebo supplementation in young adults and B) serum SOD concentrations following 2 weeks of limb immobilization in young adults. SOD, superoxide dismutase; 2 Wks. Supp., 2 weeks supplementation; LI, limb immobilization. Data are mean ± SD. Placebo n = 10, PB125 n = 10. *p < 0.05 vs baseline placebo, ^#^p < 0.05 vs 2 Wks. supp.

**Table 1:** Participant characteristics in older adults.

	Placebo (6M/7F)	PB125 (6M/5F)	P Value
Age, yr	68 ± 9	66 ± 6	0.519
Height, cm	170 ± 11	167 ± 12	0.523
Weight, kg	83 ± 17	79 ± 18	0.508
BMI, kg/m^2^	29 ± 4	28 ± 4	0.636
Resting HR, bpm	64 ± 8	65 ± 12	0.731
Brachial SBP, mmHg	124 ± 16	126 ± 13	0.726
Brachial DBP, mmHg	76 ± 8	77 ± 10	0.759
Brachial PP, mmHg	48 ± 11	49 ± 11	0.827
Brachial MAP, mmHg	92 ± 11	94 ± 10	0.722
PA, average steps/day	5,318 ± 3,966	4,959 ± 1,977	0.788
Sedentary time, min/day	1,264 ± 66	1,255 ± 72	0.778
Light PA time, min/day	137 ± 59	132 ± 61	0.855
Moderate PA time, min/day	28 ± 27	29 ± 15	0.891
Vigorous PA time, min/day	0.7 ± 1.2	0.4 ± 0.4	0.423
Blood Chemistry			
Total Cholesterol, mg/dL	190 ± 26	185 ± 45	0.721
Triglycerides, mg/dL	119 ± 41	98 ± 36	0.215
LDL-C, mg/dL	121 ± 23	121 ± 41	0.955
HDL-C, mg/dL	52 ± 11	51 ± 9	0.730
Glucose, mg/dL	92 ± 7	93 ± 10	0.765
Hematocrit, %	44 ± 4	45 ± 3	0.412
Hemoglobin, g/dL	15 ± 2	15 ± 1	0.488
Comorbidities			
Obesity, *n* (%)	6 (46)	3 (27)	-
Diabetes, *n* (%)	2 (15)	3 (27)	-
Hypertension, *n* (%)	4 (31)	3 (27)	-
Hyperlipidemia, *n* (%)	4 (31)	4 (36)	-

BMI, body mass index; HR, heart rate; SBP, systolic blood pressure; DBP, diastolic blood pressure; PP, pulse pressure; MAP, mean arterial pressure; LDL-C, low-density lipoprotein cholesterol; HDL-C, high density lipoprotein cholesterol; PA, physical activity. Data are mean ± SD.

**Table 2:** Participant characteristics in young adults.

	Placebo (4M/6F)	PB125 (4M/6F)	P Value
Age, yr	27 ± 7	28 ± 7	0.661
Height, cm	173 ± 8	171 ± 10	0.604
Weight, kg	74 ± 12	74 ± 15	0.995
BMI, kg/m^2^	24 ± 3	25 ± 5	0.672
Resting HR, bpm	73 ± 6	68 ± 14	0.309
Brachial SBP, mmHg	116 ± 9	114 ± 10	0.684
Brachial DBP, mmHg	74 ± 5	71 ± 8	0.425
Brachial PP, mmHg	42 ± 7	43 ± 6	0.780
Brachial MAP, mmHg	88 ± 6	85 ± 8	0.466
PA, average steps/day^a^	6,051 ± 2,820	6,739 ± 2,192	0.563
Sedentary time, min/day	1,248 ± 81	1,227 ± 60	0.511
Light PA time, min/day	132 ± 65	143 ± 52	0.681
Moderate PA time, min/day	29 ± 16	39 ± 18	0.180
Vigorous PA time, min/day	1.2 ± 1.0	2.0 ± 2.9	0.479
Blood Chemistry			
Total Cholesterol, mg/dL	167 ± 40	167 ± 43	0.999
Triglycerides, mg/dL	79 ± 34	72 ± 52	0.696
LDL-C, mg/dL	108 ± 40	104 ± 48	0.834
HDL-C, mg/dL	49 ± 13	58 ± 18	0.219
Glucose, mg/dL	86 ± 5	89 ± 7	0.211
Hematocrit, %	44 ± 2	44 ± 3	0.917
Hemoglobin, g/dL	15 ± 2	15 ± 1	0.249

a n = 9, BMI, body mass index; HR, heart rate; SBP, systolic blood pressure; DBP, diastolic blood pressure; PP, pulse pressure; MAP, mean arterial pressure; LDL-C, low-density lipoprotein cholesterol; HDL-C, high density lipoprotein cholesterol; PA, physical activity. Data are mean ± SD.

**Table 3:** Flow-mediated dilation characteristics in older adults.

Brachial Artery	Placebo			PB125		
	Baseline	2 Wks. Supp	Bedrest	Baseline	2 Wks. Supp	Bedrest
**FMD/Shear, AU**	0.10 ± 0.05	0.15 ± 0.13	0.07 ± 0.05	0.11 ± 0.10	0.18 ± 0.22	0.10 ± 0.07
**Baseline Diameter, mm**	4.36 ± 0.73	4.38 ± 0.68	4.35 ± 0.69	4.54 ± 0.88	4.45 ± 0.76	4.40 ± 0.73
**Peak Diameter, mm**	4.52 ± 0.76	4.55 ± 0.71	4.44 ± 0.71	4.67 ± 0.91	4.62 ± 0.78	4.56 ± 0.76
**Change in Diameter, mm**	0.17 ± 0.08	0.17 ± 0.07	0.09 ± 0.04^a,b^	0.13 ± 0.08	0.17 ± 0.06^a^	0.16 ± 0.07^a^
**Sum of Shear, AU**	54,417 ± 15,702	55,028 ± 21,529	58,665 ± 17,750	57,791 ± 19,121	60,271 ± 20,495	61,337 ± 24,908
**RH AUC**	463.7 ± 229.9	488.9 ± 281.3	447.5 ± 187.8	557.8 ± 228.1	567.2 ± 242.5	524.1 ± 164.7
**Time to peak, s**	56 ± 5	59 ± 9	62 ± 8	54 ± 9	56 ± 10	56 ± 10
Popliteal Artery	Placebo			PB125		
	Baseline	2 Wks. Supp	Bedrest	Baseline	2 Wks. Supp	Bedrest
**FMD/Shear, AU**	−0.5 ± 2.3	0.4 ± 0.6	0.4 ± 1.0	0.3 ± 0.4	0.4 ± 0.4	0.9 ± 1.3
**Baseline Diameter, mm**	7.02 ± 0.89	7.02 ± 0.77	6.93 ± 0.75	7.30 ± 0.80	6.97 ± 1.06	6.92 ± 1.10
**Peak Diameter, mm**	7.12 ± 0.90	7.12 ± 0.83	6.97 ± 0.77	7.60 ± 0.89	7.13 ± 1.08	7.10 ± 1.11
**Change in Diameter, mm**	0.10 ± 0.09	0.10 ± 0.09	0.04 ± 0.06	0.31 ± 0.62	0.16 ± 0.10	0.18 ± 0.09
**Sum of Shear, AU**	17,932 ± 12,235	15,871 ± 6,944	12,870 ± 5,311	21,229 ± 14,743	22,674 ± 16,462	14,505 ± 10,943^a,b^
**RH AUC**	545.5 ± 330.1	488.8 ± 204.6	412.1 ± 140.8	707.5 ± 313.3	675.4 ± 257.6	779.4 ± 917.4
**Time to peak, s**	54 ± 11	55 ± 10	74 ± 11	52 ± 14	69 ± 11	62 ± 14

RH, reactive hyperemia; s, seconds; supp, supplement; Wk. week(s).

a p<0.05 vs Baseline within group.

b p < 0.05 vs Wks. Supp. within group.

**Table 4:** Flow-mediated dilation characteristics in younger adults.

Brachial Artery	Placebo			PB125		
	Baseline	2 Wks. Supp	2 Wks. LI	Baseline	2 Wks. Supp	2 Wks. LI
**FMD/Shear, AU**	0.13 ± 0.05	0.16 ± 0.15	0.13 ± 0.04	0.14 ± 0.05	0.23 ± 0.34	0.32 ± 0.48
**Baseline Diameter, mm**	3.87 ± 0.55	3.81 ± 0.48	3.85 ± 0.66	4.03 ± 0.56	3.93 ± 0.44	4.10 ± 0.51
**Peak Diameter, mm**	4.11 ± 0.60	4.03 ± 0.51	4.08 ± 0.72	4.28 ± 0.57	4.12 ± 0.43	4.10 ± 0.51
**Change in Diameter, mm**	0.24 ± 0.09	0.22 ± 0.08	0.24 ± 0.08	0.25 ± 0.10	0.20 ± 0.08	0.21 ± 0.13
**Sum of Shear, AU**	71,570 ± 22,511	73,419 ± 18,561	71,949 ± 17,930	68,305 ± 34,324	70,607 ± 49,792	74,959 ± 37,794
**RH AUC**	474.7 ± 256.2	435.7 ± 146.9	457.6 ± 238.8	453.8 ± 156.9	397.2 ± 182.2	499.8 ± 188.8
**Time to peak, s**	54 ± 6	56 ± 8	56 ± 7	57 ± 8	62 ± 12	62 ± 11
Popliteal Artery	Placebo			PB125		
	Baseline	2 Wks. Supp	2 Wks. LI	Baseline	2 Wks. Supp	2 Wks. LI
**FMD/Shear, AU**	0.33 ± 0.38	0.32 ± 0.42	0.11 ± 0.08	0.23 ± 0.22	0.61 ± 0.87^a^	0.20 ± 0.15^b^
**Baseline Diameter, mm**	5.14 ± 0.94	5.36 ± 0.82	4.97 ± 0.66^b^	5.28 ± 0.69	5.16 ± 0.66	5.04 ± 0.79^a^
**Peak Diameter, mm**	5.44 ± 0.98	5.62 ± 0.89	5.10 ± 0.66^a,b^	5.45 ± 0.65	5.38 ± 0.62	5.20 ± 0.82
**Change in Diameter, mm**	0.33 ± 0.15	0.26 ± 0.17	0.12 ± 0.08^a,b^	0.17 ± 0.14	0.22 ± 0.13	0.17 ± 0.09
**Sum of Shear, AU**	29,448 ± 8,208	29,929 ± 4,988	35,533 ± 15,999^a^	26,241 ± 8,238	22,865 ± 10,437	29,266 ± 10,094^b^
**RH AUC**	477.2 ± 346.9	509.2 ± 224.3	443.6 ± 271.8	378.6 ± 118.1	296.9 ± 128.5^c^	350.2 ± 158.8
**Time to peak, s**	69 ± 9	60 ± 12	53 ± 8	72 ± 12	49 ± 12	78 ± 9

RH, reactive hyperemia; s, seconds; supp, supplement; LI, limb immobilization; Wks., weeks.

a p<0.05 vs Baseline within group.

b p < 0.05 vs 2 Wks. Supp. within group.

c p < 0.05 vs Placebo.

**Table 5:** Leg blood flow and vascular conductance during passive leg movement in older adults.

	Placebo			PB125		
	Baseline	2 Wks. Supp	Bedrest	Baseline	2 Wks. Supp	Bedrest
LBF, mL•min^−1^						
**Baseline**	311 ± 122	320 ± 113	274 ± 72^b^	288 ± 99	273 ± 97	252 ± 68
**Peak**	712 ± 233	741 ± 241	627 ± 166^a,b^	593 ± 294	620 ± 298	584 ± 276
**Delta Δ**	401 ± 141	422 ± 171	353 ± 127^b^	304 ± 240	347 ± 241	332 ± 234
LVC, mL•min^−1^• mmHg						
**Baseline**	3.4 ± 1.4	3.5 ± 1.2	3.0 ± 0.7^b^	3.2 ± 1.1	3.0 ± 1.2	2.8 ± 0.7
**Peak**	7.7 ± 2.7	8.1 ± 2.6	6.8 ± 1.7^b^	6.3 ± 3.3	6.7 ± 3.3	6.8 ± 3.8
**Delta Δ**	4.3 ± 1.6	4.6 ± 1.9	3.8 ± 1.3	3.2 ± 2.6	3.7 ± 2.6	3.9 ± 3.3

LBF, leg blood flow; LVC, leg vascular conductance; Supp, supplement; Wks. weeks.

a p<0.05 vs Baseline within group.

b p < 0.05 vs 2 Wks. Supp. within group.

**Table 6:** Leg blood flow and vascular conductance during passive leg movement in younger adults.

	Placebo			PB125		
	Baseline	2 Wks. Supp	2 Wks. LI	Baseline	2 Wks. Supp	2 Wks. LI
LBF, mL•min^−1^						
**Baseline**	334 ± 144	322 ± 83	342 ± 150	308 ± 126	263 ± 78	246 ± 79
**Peak**	866 ± 260	833 ± 276	760 ± 226	718 ± 277	700 ± 310	647 ± 270
**Delta Δ**	531 ± 241	510 ± 262	419 ± 243	410 ± 247	436 ± 310	400 ± 242
LVC, mL•min^−1^• mmHg						
**Baseline**	3.8 ± 1.6	3.6 ± 0.8	3.8 ± 1.7	3.7 ± 1.5	3.1 ± 1.0	2.8 ± 0.8
**Peak**	9.9 ± 2.5	9.6 ± 3.1	8.5 ± 2.6	8.5 ± 3.1	8.3 ± 3.6	7.2 ± 2.8
**Delta Δ**	6.1 ± 2.4	6.0 ± 2.9	4.7 ± 2.7	4.8 ± 2.6	5.2 ± 3.6	4.4 ± 2.6

LBF, leg blood flow; LVC, leg vascular conductance; Supp, supplement; LI, limb immobilization; Wks. weeks.

**Table 7:** Central hemodynamics during passive leg movement in older adults.

	Placebo			PB125		
	Baseline	2 Wks. Supp	Bedrest	Baseline	2 Wks. Supp	Bedrest
CO, L/min						
**Baseline**	4.9 ± 1.5	5.2 ± 1.9	5.0 ± 1.2	4.8 ± 1.4	5.1 ± 1.4	4.3 ± 1.4
**Peak**	5.5 ± 1.7	5.9 ± 2.1	5.5 ± 1.4	5.3 ± 1.5	5.7 ± 1.6	4.9 ± 1.7
**Delta Δ**	0.6 ± 0.3	0.7 ± 0.2	0.6 ± 0.3	0.5 ± 0.3	0.6 ± 0.3	0.6 ± 0.4
SV, mL/beat						
**Baseline**	75 ± 27	84 ± 34	73 ± 21	77 ± 26	85 ± 32	75 ± 19
**Peak**	82 ± 30	92 ± 37	82 ± 30	83 ± 27	92 ± 33	83 ± 27
**Delta Δ**	7 ± 5	8 ± 4	10 ± 23	6 ± 5	7 ± 4	8 ± 26
MAP, mmHg						
**Baseline**	92 ± 10	91 ± 9	92 ± 7	93 ± 11	89 ± 10	91 ± 7
**Peak**	100 ± 9	100 ± 11	100 ± 6	102 ± 10	100 ± 10	100 ± 6
**Delta Δ**	8 ± 5	9 ± 4	8 ± 4	9 ± 4	11 ± 17	10 ± 10
HR, bpm						
**Baseline**	64 ± 8	64 ± 10	71 ± 8^a,b^	66 ± 12	63 ± 13	71 ± 15^a,b^
**Peak**	68 ± 7	69 ± 9	75 ± 8^a,b^	72 ± 13	69 ± 14	78 ± 15^a,b^
**Delta Δ**	5 ± 3	5 ± 2	5 ± 3	6 ± 6	6 ± 5	6 ± 4

CO, cardiac output; SV, stroke volume; MAP, mean arterial pressure; HR, heart rate. Supp, supplement; Wks. weeks.

a p<0.05 vs Baseline within group.

b p < 0.05 vs 2 Wks. Supp. within group.

**Table 8:** Central hemodynamics during passive leg movement in younger adults.

	Placebo			PB125		
	Baseline	2 Wks. Supp	2 Wks. LI	Baseline	2 Wks. Supp	2 Wks. LI
CO, L/min						
**Baseline**	5.7 ± 1.1	5.8 ± 1.1	6.1 ± 0.8	5.5 ± 1.3	5.4 ± 1.0	5.8 ± 1.1
**Peak**	6.6 ± 1.4	6.4 ± 1.0	6.9 ± 0.8	6.1 ± 1.4	6.0 ± 1.0	6.5 ± 1.3
**Delta Δ**	0.9 ± 0.5	0.6 ± 0.3	0.8 ± 0.3	0.5 ± 0.3^a^	0.6 ± 0.3	0.7 ± 0.3
SV, mL/beat						
**Baseline**	80 ± 16	74 ± 11	76 ± 10	82 ± 22	85 ± 20	87 ± 17
**Peak**	87 ± 14	83 ± 14	83 ± 10	89 ± 22	92 ± 20	94 ± 20
**Delta Δ**	7 ± 3	9 ± 10	6 ± 4	6 ± 2	7 ± 4	7 ± 5
MAP, mmHg						
**Baseline**	88 ± 6	89 ± 6	89 ± 4	84 ± 8	84 ± 6	88 ± 5^b^
**Peak**	94 ± 6	96 ± 8	95 ± 6	91 ± 9	92 ± 8	95 ± 5 ^b,c^
**Delta Δ**	6 ± 4	7 ± 4	6 ± 3	6 ± 1	7 ± 3	7 ± 2
HR, bpm						
**Baseline**	73 ± 6	76 ± 8	78 ± 5^a^	66 ± 9	65 ± 7^d^	69 ± 10^e,b^
**Peak**	79 ± 6	84 ± 9^a^	86 ± 7^a^	70 ± 9^a^	71 ± 8^d^	74 ± 7^e^
**Delta Δ**	6 ± 3	8 ± 4	8 ± 4	4 ± 3	6 ± 4	5 ± 2

CO, cardiac output; SV, stroke volume; MAP, mean arterial pressure; HR, heart rate. Supp, supplement; LI, limb immobilization; Wks. weeks.

a p<0.05 vs Placebo Baseline.

b p < 0.05 vs 2 Wks. Supp. within group.

c p < 0.05 vs Baseline within group.

d p < 0.05 vs Placebo 2 Wks. Supp.

e p < 0.05 vs Placebo 2 Wks. LI.

## Data Availability

Data will be made available upon reasonable request.
